# Women born with very low birth weight have similar menstrual cycle pattern, pregnancy rates and hormone profiles compared with women born at term

**DOI:** 10.1186/s12905-019-0753-y

**Published:** 2019-04-25

**Authors:** Gunilla Sydsjö, Pia Törnblom, P-O Gäddlin, Orvar Finnström, Ingemar Leijon, Nina Nelson, Elvar Theodorsson, Mats Hammar

**Affiliations:** 1 Department of Obstetrics and Gynecology, Department of Clinical and Experimental Medicine, Linköping University, SE-58185 Linköping, Sweden; 2 Department of Pediatrics, Department of Clinical and Experimental Medicine, Linköping University, SE-58185 Linköping, Sweden; 3 Department of Quality and Patient Safety, Karolinska University Hospital, Stockholm, SE-17176 Sweden; 4 Clinical Chemistry, Department of Clinical and Experimental Medicine, Faculty of Medicine and Health Sciences, Linköping University, SE-58185 Linköping, Sweden

**Keywords:** Infertility, Ovarian function, Menstrual cycle, Preterm

## Abstract

**Background:**

Individuals born very preterm or with very low birth weight (VLBW) have a reduced likelihood to reproduce according to population-based register studies. Extremely low-birth weight born adults had a lower reproduction rate for both men and women in a follow-up study.

**Aim:**

To investigate if being born with VLBW is associated with differences in the reproductive health, i.e. age of menarche, menstrual cycle pattern, pregnancy rates and hormone profile compared with women born at term.

**Methods:**

A prospective long-term follow-up of a cohort of live-born VLBW children and their controls studied repeatedly since birth and now assessed at 26–28 years of age. Of the totally 80 girls enrolled from birth 49 women (24 VLBW women and 25 controls) participated in the current follow-up. The women’s anthropometric data and serum hormone levels were analysed.

**Results:**

The reproductive hormone levels, including Anti-Mullerian Hormone, did not differ significantly between VLBW women and their controls. Both groups reported menstrual cycle irregularities and pregnancies to the same extent but the VLBW women reported 1.5 years later age of menarche. The VLBW subjects had a catch-up growth within 18 months of birth but remained on average 5 cm shorter in adult height. There were no significant differences in BMI, sagittal abdominal diameter, blood pressure or in their answers regarding life style between the VLBW women and the controls.

**Conclusion:**

No differences in the reproductive hormone levels were found between VLBW women and their controls. Although age at menarche was somewhat higher in the VLBW group menstrual cycles and pregnancy rates were similar in the VLBW and control groups. Further follow-up studies are required to elucidate the health outcomes of being born VLBW.

## Background

Medical advances in perinatal medicine and neonatal intensive care have considerably improved the survival rates for preterm births. Today infants born before week 24 and weighing less than 500 g may survive [1–3]. In Sweden in 2014 around 6% of all births were preterm, i.e. < 37 weeks gestational age [4] and 0.5% were born with a weight < 1500 g, i.e. very low birth weight (VLBW). The neonatal mortality in Sweden (within 27 days postpartum) for preterm births (< 37 weeks) has declined from 4.2% in 1985 to 2.0% in 2014, and for children born before 28 weeks gestational age from 47 to 19% respectively [4].

Several long-term follow-up studies of individuals born VLBW have assessed how birth size and gestational age affect their health in child- and adulthood. A meta-analysis from 2010 showed an inverse association between birth weight and all-cause adult mortality [5].

The effects of intrauterine programming on the development of the reproductive function including ovarian development of preterm and small for gestational age infants have not yet been elucidated. Population-based register studies from Sweden and Norway show that individuals born very preterm or with VLBW have a reduced likelihood to reproduce [6, 7] In a recent follow-up, study the extremely low-birth weight born adults showed a lower reproduction rate for both men and women compared with normal-weight born adults investigated at age 29–36 years. The men and women were also less likely to be in a relationship or having had sexual intercourse and a higher proportion reported ill health and more chronic health conditions [8].

The development of the hypothalamus-pituitary-gonadal-axis (HPG-axis) in preterm infants is not fully understood but the literature does contain theories of maldevelopment. VLBW infant girls have 10–20 times higher follicle stimulating hormones (FSH) levels and 3–4 times higher luteinizing hormones (LH) than controls born at term the first 10 weeks after delivery. Consequences of this exaggerated activation of the HPG-axis have not been elucidated in humans, but in monkeys this exaggerated postnatal rise in FSH has been seen to correlate with HPG-axis disorders in the pubertal period [9].

An association has been found between insulin resistance and birth weight in VLBW as well as low birth weight (LBW) young adults [10]. In young male- and female adults born preterm there was a negative association between birth weight and testosterone [11]. A possible mechanism for the increased androgen levels is that insulin may increase the synthesis of androgens within the theca cell irrespective of the LH level [12]. High androgen levels are also associated with polycystic ovarian syndrome (PCOS), which is more prevalent in LBW females [10].

The objective of this prospective controlled cohort study with follow-up at ages 27 to 28 years, was to investigate if being born with VLBW was associated with differences in the reproductive health, i.e. age of menarche, menstrual cycle pattern, pregnancy rates, and hormone profile compared with women born at term.

## Methods

### Study population

A cohort of live-born VLBW children in the Southeast region of Sweden has participated in follow-up studies from birth to the present study at ages 27–28 years. Previous follow-up investigations have been made 6 and 18 months after birth and at 4, 9, 12, 15, and 20 years of age. All children born VLBW and treated in neonatal units in the South-East region of Sweden (59% in Linköping University Hospital) and born between 1 February 1987 and 30 April 1988, were initially included in the study, in total 107 children to 97 mothers. They represented 0.72% of births in the region during this period. Out of the 107 children born VLBW 47 were girls and 39 of them survived their first month. One child with Down’s syndrome was excluded from follow up and thus 38 girls with VLBW were followed. One girl born at term, at the same hospital (or at the hospital where she would have been born, if the mother had not been referred before birth), without malformation, with the same parity and born next in order after each VLBW child was selected as a control. The control group consisted of 41 girls. One of the VLBW girls (2.6%) was a twin and none of the control children was a twin. Among the VLBW girls 27 (71%) were born small for gestational age (SGA) and 8 (21%) were born with extremely low birth weight (ELBW), i.e. born below 1000 g. Two of the VLBW girls were diagnosed with cerebral palsy (CP). The mean birth weight was 1169 g (SD 212 g) for the VLBW girls and 3400 g (SD 561 g) for the girl controls. The lightest surviving girl had a birth weight of 740 g. The mean gestational age was 31 weeks (SD 2.8) for the VLBW girl survivors with a range between 25 and 37 weeks.

All of the 79 women (38 girls with VLBW, the index group, and 41 female controls) from the original cohort still living in Sweden were contacted by mail with detailed information on the study. Letters of reminder were sent to the non-responders. Written informed consent was obtained from all study participants. Questionnaires were answered at home and the subjects were asked to choose a day convenient to them for examinations and blood sampling at the paediatric clinic. Two research nurses and two medical students carried out the examinations. Subjects who were unable or unwilling to come to any of the hospitals were asked to participate by answering the questionnaires and by visiting their primary health care centre for blood sampling. In all, 49 women (24 index and 25 controls) agreed to participate in the study.

**Fig. 1:**
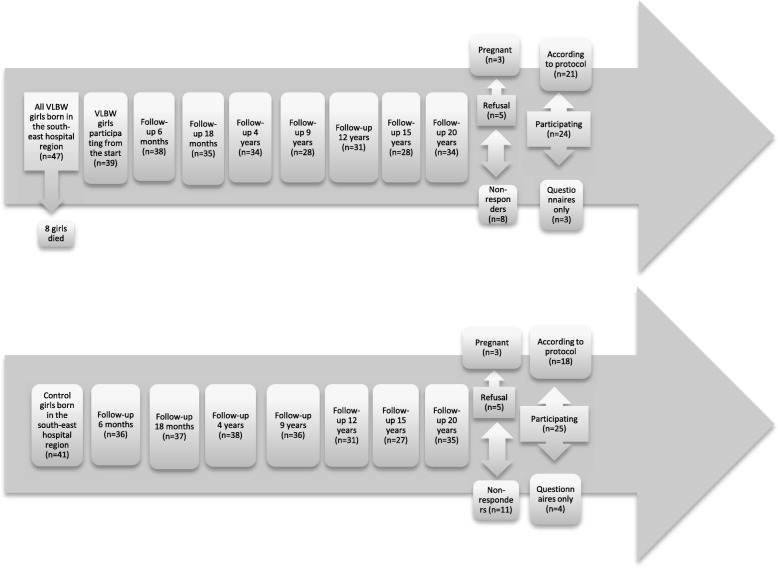
Flowcharts showing the number of participants in two groups of girls born either with Very Low Birth Weight (VLBW) or at term (controls) in the follow-ups since the start in 1987

Serum samples and anthropometric measurements were not taken from nine women who were pregnant or newly delivered. One from the index group and five controls only answered the questionnaires. Twenty-five of the 49 women were currently using hormonal contraceptives at the time of blood sampling and were therefore excluded from the serum sample analyses of LH, FSH, estradiol, testosterone and sex hormone binding globulin (SHBG). Nor was Anti-Mullerian Hormone (AMH) analysed from these women. I.e. neither sex hormone levels nor menstrual cycle data were analysed on women using hormonal contraceptives. Examinations took place from April 2014 until May 2015. Figure 1 is a flowchart showing the number of participants in the two groups in the follow-ups since the start in 1987.

#### Questionnaires

The study-specific questionnaire for women asked for lifestyle factors including civil status, form of housing, education, occupation, family, health and activities, questions concerning reproductive health, concerning gynaecological conditions, contraceptive use, age at menarche, pregnancies, abortions, miscarriages, menstrual irregularities during the last 6 months, and date of the last menstrual bleeding. Women using oral contraceptives (*n* = 25) were asked about possible menstrual irregularities before they started with the oral contraceptives. Menstrual regularity was defined as a menstrual cycle length of 24–35 days.

#### Hormone analyses

Blood samples were obtained regardless of the day in their menstrual cycle when they visited the hospital. Date for last menstrual bleeding was asked for as well as hormonal contraceptive use and menstrual cycle irregularities. FSH and LH concentrations were measured using Immulite 2000 XPi (Siemens diagnostics). Estradiol, total testosterone and SHBG were analyzed using Cobas e602 (Roche Diagnostics). Serum TSH, free T4 and free T3 were all analyzed using ADVIA Centaur XP (Siemens diagnostics). AMH measurement was preformed using an ELISA (AnshLabs, Webster, USA).

### Statistical analysis

All statistical analyses were performed using Statistical Package for the Social Sciences (SPSS) version 23 for Mac (IBM Corp., Armonk, NY). Each quantitative variable was expressed as mean, median, interquartile range and standard deviation. The Kolmogorov-Smirnov test was used to test the normality of the distribution of the hormone samples. Owing to the limited number of observations and skewed distribution non-parametric tests were used. Differences by means between the VLBW women and women in the control group were analysed with Mann-Whitney U-tests performed for all hormone samples, anthropometric measurements and age at menarche.. A Pearson product-moment correlation coefficient was computed to assess the bivariate relationship between the BMI) and menarche. The proportional differences for qualitative variables such as menstrual regularity and use of hormonal contraceptives were tested with Chi-square test. A (two-sided) *p*-value < 0.05 was considered statistically significant.

## Results

From the totally 80 girls enrolled at birth, 24 VLBW women and 25 controls participated in this follow-up (response rate 61%). Out of the 39 women who did not participate in the follow-up, one had moved abroad, 10 women expressed that they did not wish to participate while 28 women did not participate for unknown reasons (non-responders). Eight women were pregnant and one newly delivered and thus participated by completing questionnaires only. In addition, 25 of the 49 women (51%) were using hormonal contraceptives, which excluded them from some of the statistical analyses since their contraception affected their serum hormone levels. Demographics and self-reported health data are presented in Table 1, showing no differences between the groups. Also, among the participating women 19 (79.2%) of the 24 VLBW women were born SGA.

**Table 1:** Demographic and self-reported health data for the two groups of women

	VLBW (*n* = 24)		Control (*n* = 25)		
	n	%	n	%	*p*-value
Marital status					0.682
Single	7	29.2	6	24.0	
Partner (married/cohabiting)	17	70.8	19	76.0	
Living arrangements					0.495
Rental	16	66.7	12	50.0	
Condo	3	12.5	5	20.8	
Single family house	5	20.8	7	29.2	
Employed					0.470
Yes (full & part time)	16	66.7	19	76.0	
No (unemployed, sick-leave, student)	8	33.3	6	24.0	
Educational level					0.611
Elementary	1	4.3	2	8.0	
Secondary	16	69.6	14	56.0	
University	6	26.1	9	36.0	
Have children					1.000
No	17	70.8	17	70.8	
Yes	7	29.2	7	29.2	
Desire to have children					0.975
No	1	4.3	1	4.2	
Yes	22	95.7	23	95.8	
Self-reported good health					0.637
No	2	8.3	3	12.5	
Yes	22	91.7	21	87.5	
Regular use medication other than contraceptives					0.353
No	15	65.2	13	52.0	
Yes	8	34.8	12	48.0	
Tobacco use					0.667
No	15	65.2	16	64.0	
Snuff	3	13.0	2	8.0	
Cigarettes	5	21.7	5	20.0	
Both	0	0.0	2	8.0	
Alcohol use					0.365
Never	4	17.4	4	17.4	
1–4 times a month	11	47.8	9	36.0	
2–3 times a week	8	34.8	12	48.0	
Exercise					0.459
Never	1	4.3	0	0.0	
0–1 times a week	5	21.7	5	20.0	
2–4 times a week	11	47.8	9	36.0	
Almost every day	6	26.1	11	44.0	
History of Gynaecological problems					0.979
No	12	54.5	13	54.2	
Yes	10	45.5	11	45.8	
History of miscarriage					0.438
No	9	75.0	13	86.7	
Yes	3	25.0	2	13.3	
History of induced abortion					0.409
No	6	46.2	11	61.1	
Yes	7	53.8	7	38.9	
Regular menstrual periods prior to oral contraceptives					1.000
No	1	0.12	1	0.12	
Yes	7	0.78	7	0.78	

Table 2 shows the birth characteristics, anthropometric data and blood pressures collected. The VLBW subjects had a significantly lower birth weight than controls. There was a 9 weeks difference in median gestational age between groups. Totally 69% of the VLBW girls were born SGA. At age 27–28 years, there was still a significant difference in height between index and controls, the index women being about 5 cm shorter (*P* = 0.010). BMI, sagittal abdominal diameter (SAD) and systolic as well as diastolic blood pressure did not differ between the groups. However, VLBW women seem to have a somewhat higher diastolic blood pressure compared to control women (*p* = 0.047).

**Table 2:** Birth characteristics, clinical anthropometrical data and blood pressures of all women in the VLBW and control group (median and interquartile range)

	VLBW	Control	*p*-value
Gestational age (weeks)	32 (4)	39 (2)	< 0.001
SGA (%)	69	0	< 0.001
Birth weight (kg)	1.179 (0.342)	3.610 (0.755)	< 0.001
Height (m)	1.61 (0.06)	1.65 (0.07)	0.023
BMI (kg/m^2^)	21.81 (2.12)	24.57 (5.06)	0.072
Sagittal abdominal diameter (cm)	17.60 (3.0)	19.50 (3.4)	0.253
Systolic blood pressure (mmHg)	122.0 (11.17)	114.0 (17.17)	0.047
Diastolic blood pressure (mmHg)	78.7 (10.67)	71.0 (11.58)	0.066

Table 3 shows the serum hormonal levels. Only nine subjects and seven controls were not using hormonal contraceptives or were not pregnant and could therefore be included in the hormonal analyses. There were no significant differences between VLBW subjects and controls in any measured hormonal levels. All hormone levels were within reference limits for both groups, except for one testosterone sample of 2.2 nmol/L from a VLBW subject, one elevated estradiol sample of 1460 pmol/L in the control group and two AMH values at 7.1 and 14.2 μg/L in the control group. The birth weight of the VLBW subjects participating with hormone samples (median 1.209 kg) did not differ significantly from the VLBW subjects who did not participate (median 1.223 kg, *p*-value 0.765).

**Table 3:** Hormone level comparisons between VLBW and control group. Results presented as medians (interquartile range)

Hormone	Reference interval	VLBW (*n* = 9)	Controls (*n* = 7)	*p*-value
LH (IE/L)	1.0–96	3.9 (4.3)	4.0 (13.2)	0.739
FSH (IE/L)	1.7–22	4.2 (4.1)	3.4 (3.2)	0.183
Testosterone (nmol/L)	< 1.8	0.7 (0.9)	0.8 (1.0)	0.827
SHBG (nmol/L)	19–145	67.0 (44.0)	88.0 (94.5)	0.219
Testosterone/SHBG ratio	< 0.07	0.01 (0.02)	0.01 (0.01)	0.974
Estradiol (pmol/L)	90–1160	260.0 (459.8)	380.0 (927.0)	0.377
AMH (μg/L)	0.7–6	2.4 (2.9)	2.4 (4.4)	0.826
TSH (mIE/L)	0.4–4	1.6 (1.2)	1.2 (1.1)	0.148
T3 (pmol/L)	3.5–6	4.9 (0.8)	4.9 (0.4)	0.741
T4 (pmol/L)	0.11–22	15.4 (2.2)	15.1 (2.9)	0.269

The women were living in couple relationships and reported good self-perceived health to the same extent in both groups (Table 4). No significant difference was found in the number of women reporting regular menstrual cycles or if they ever had been pregnant. Recall data on age at menarche showed significant differences, the VLBW group reporting menarche on average about 1.5 years later than the women in the control group (*p* = 0.016). The proportion of women using hormonal contraceptives at the time of follow-up was equal in both groups.

**Table 4:** Fertility related questions. Selected variables from women’s lifestyle questionnaires. Absolute numbers given within brackets (except for “Age at menarche” which is interquartile range)

	VLBW	Controls	*p*-value
Age at menarche (years)^a^	13.5 (2.25)	12.0 (1.25)	0.016
Regular menstrual cycle, % (n)	45.8 (11)	52.9 (9)	0.492
Hormonal contraceptives, % (n)	54.2 (13)	48.0 (12)	0.669
Good health, % (n)	91.7 (22)	87.5 (21)	0.640
Couple relationship, % (n)	70.8 (17)	76.0 (19)	0.685
Pregnancies, % (n)	64.7 (11)	65.0 (13)	0.985

^a^Data expressed as median (interquartile range)

A Pearson product-moment correlation coefficient was computed to assess the relationship between the BMI at age 9 years (data not shown) and menarche in the VLBW group. There was a moderate, negative correlation between the two variables, r = − 0.533, *n* = 24, *p* = 0.001 indicating that a lower BMI in childhood was correlated with older age at menarche.

## Discussion

We found no differences between the VLBW group and the control group born at term in reproductive hormone levels, menstrual cycle irregularities, pregnancy rates or reproductive hormone profiles, whereas the VLBW group reported menarche about 1.5 years later than the control women.

A strength of this study was that the participants constituted a well-defined cohort of VLBW girls matched at birth with controls born at term, at the same hospital, with the same sex and mother’s parity to reduce the number of possible confounders and socio-economic inequalities. The study population was from the southeast region of Sweden where the level of neonatal as well as maternal care should have been relatively equal from hospital to hospital in 1987–88. The women had been participating in several follow-up examinations since birth, mostly regarding neurodevelopmental outcomes and school achievements. An important feature when attempting to evaluate the long-term health effects of being born with VLBW is the characteristics of the subjects participating in the study. At enrollment to this study the parent’s decision gave a high participation rate for both well and unwell infants. At the present follow up the young women were not as willing to participate causing a risk for selection bias of more healthy individuals. Nevertheless, the participation rate and number of non-responders did not differ between the VLBW and the control group. Also, the birth weight of the VLBW women participating did not differ significantly from the VLBW women not participating, suggesting that we did not have a selection of only the healthiest women in the VLBW group.

Due to the long-term character of this study a considerable dropout was not unexpected (response rate was 56%) but leads to a low power well below 50%. This jeopardizes the generalizability and interpretations of the results. Another limitation was that many women, as expected in this age group, used hormonal contraceptives or were pregnant making hormone analyses less interpretable. Therefore, we can neither dismiss nor confirm that abnormal reproductive hormone profiles were more prevalent in the VLBW group than in the controls.

For the convenience of the participants the blood samples were taken regardless of the day in the menstrual cycle. Ideally all samples should have been drawn at more than one time point, such as at the time of ovulation and during the midluteal phase but that was not possible due to practical reasons and long distance from the hospital for many participants. Since the hormone levels vary depending of the phase of the menstrual cycle, the FSH, LH and estradiol levels were difficult to compare. Even though the date of the last menstrual bleeding was asked for, the LH, FSH and estradiol samples were too few in each group to enable categorizing them as normal with regard to menstrual cycle phase. AMH levels were similar in the two groups as judged by the limited number of AMH samples. Unfortunately we did not analyse AMH levels in women using oral contraceptives because we omitted all hormonal analyses in these women, but AMH is probably not affected by use of oral contraceptives and could have added some information.

AMH has been studied before in small groups but the results are disparate [13–16]. One study showed no significant difference in AMH levels between women born preterm or SGA and women born with normal birth weight except for the subgroup with a rapid catch-up growth where the AMH levels were higher [15]. We were, unfortunately, unable to compare individuals with and without rapid catch-up growth because of the small number of participants.

Swedish and Norwegian register studies have shown that low birth weight is associated with reduced parity in line with results from a resent follow up from Canada on 60 women and 40 men who were born with ELBW [6–8]. This could evidently be related to other factors than low fertility and ovulatory dysfunction. However, in our study we found similar pregnancy rates. Cohabitation and self-perceived good health were also equal in both groups. It could be argued that reproductive hormones are not a valid measure of fertility and the number of pregnancies is more relevant. The mean age at the first delivery is 28 years in the south-east region of Sweden and ideally the subjects would have been older before they were studied for the number of childbirths in this cohort (2015).

When attempting to assess female fertility, ovulation is a fundamental condition for being fertile. Hormone measurements, menstrual cycle regularity, and vaginal ultrasound of the ovaries are important tools used to monitor ovulation and the menstrual cycle. The presence of menstrual disorders such as amenorrhea and oligomenorrhea often indicates an ovulatory disorder and reduced fertility. In this study we therefore attempted to compare the prevalence of irregular menstrual cycles between the two groups. We found no statistical difference regarding menstrual cycle irregularities but the data should be interpreted keeping in mind that retrospective questionnaires were used for the assessment. Furthermore, women who were using hormonal contraceptives (46% in our cohorts) were asked to recall their spontaneous menstrual cycles retrospectively before starting hormonal contraception, sometimes years earlier. Some earlier studies have attempted to collect similar observations. One review described the occurrence of reduced ovulation rates in adolescent LBW girls, however, a larger study on 704 SGA and appropriate for gestational age (AGA) women found no difference in menstrual cycle length between the two groups [17, 18]. Saigal and coworkers [8] found that women born with ELBW did not report higher incidence of menstrual irregularities than control women born at term [8].

From the questionnaires in this study we found that VLBW women were 1.5 years older at menarche than the controls. This is in contrast to a small study which found that menarche occurred 5–10 months earlier in girls born preterm and with [17], the lower the birth weight the earlier menarche. Furthermore, the LBW girls had a more rapid progression through puberty and a reduced adult height [17]. Another study found no difference in age at menarche between LBW and AGA individual [14]. Our data were collected from questionnaires from a small cohort at the age 27–28 years and there is a potential risk of recall bias. A possible explanation for our findings is the previously described inverse correlation between BMI in childhood and age at menarche [19]. We found a moderate negative correlation between the BMI at age 9 and menarche in the VLBW group and the 1.5 years older age at menarche in the VLBW group could be explained by a significantly lower BMI in childhood.

The finding that VLBW combined with SGA but not VLBW and AGA women had a lower adult BMI than controls [20], could not be confirmed in our study as we could not analyze subgroups. Another finding was that the VLBW women did not reach a catch-up in height and remained on average 5 cm shorter as adults. This is in accordance with other studies where most girls born with VLBW have a rapid catch-up growth and the disparities in height and weight have equalized within 2 years of age but they have a shorter end stature as adults [20, 21]. The reduced adult height is thought to be attributable to an abnormal pre-pubertal growth. This could be explained by the fact that the maximal growth velocity lasts for a shorter time during puberty and is reached earlier in these individuals [22].

The sagittal abdominal diameter (SAD), which is considered a good measurement of visceral adiposity and associated with increased risk of cardiovascular disease and insulin resistance, was not found to be significantly different between the two groups in our study. Other studies have found that VLBW girls have an increased amount of visceral fat and more often have insulin resistance [23]. The premature children with a rapid catch-up growth seem to be more prone to develop an adipose body composition and increased BMI as adults, with an increased risk of the metabolic syndrome [24], and elevated systolic blood pressure [25]. This could, however, not be confirmed in our small cohort of young adults. Measurements of circulating cholesterol, triglycerides and fasting plasma glucose would have been advantageous in this context.

Our study of reproductive hormone levels can be regarded as a pilot study and a larger sample size is needed. Similar VLBW cohorts participating in long-term follow-up studies exist in Norway and Finland, with similar neonatal care as in Sweden. A merging of data from the different groups born in the late 1980s could provide the basis for interesting studies with sufficient power. Also these groups should be followed throughout their reproductive time span in order to evaluate their reproductive performance.

## Conclusion

From the data in our present study we can conclude that VLBW does not seem to dramatically affect reproductive hormone levels, menstrual cycle irregularities or pregnancy rates. We found, however, postponed menarche and shorter height in the adults born with VLBW. With the constant progress in neonatal care and the possibility for more ELBW and VLBW babies to survive there is a continuing need for new long-term follow-up studies in order to identify risks which could be prevented by different measures during childhood and adolescence.

## Abbreviations

AGA: Appropriate for Gestational Age
AMH: Anti-Mullerian Hormone
BMI: Body Mass Index
CP: Cerebral Palsy
ELBW: Extremely Low Birth Weight
FSH: Follicle Stimulating Hormones
HPG: Hypothalamus-Pituitary-Gonadal
LBW: Low Birth Weight
LH: Luteinizing Hormones
PCOS: PolyCystic Ovarian Syndrome
SAD: Sagittal Abdominal Diameter
SD: Standard Deviation
SGA: Small for Gestational Age
SHBG: Sex Hormone Binding Globulin
VLBW: Very Low Birth Weight
